# Advanced materials for disease diagnostics

**DOI:** 10.1016/j.mtbio.2023.100676

**Published:** 2023-05-19

**Authors:** Lin Huang, Kun Qian

**Affiliations:** Department of Clinical Laboratory Medicine, Shanghai Chest Hospital, School of Medicine, Shanghai Jiao Tong University, Shanghai, 200030, PR China; Shanghai Institute of Thoracic Oncology, Shanghai Chest Hospital, School of Medicine, Shanghai Jiao Tong University, Shanghai, 200030, PR China; School of Biomedical Engineering, Institute of Medical Robotics and Med-X Research Institute, Shanghai Jiao Tong University, Shanghai, 200030, PR China

Early diagnosis of diseases prior to the visibility of anatomic anomalies, has been universally accepted to be essential for the success of health management. However, current medical protocols may require more than a million cells for accurate clinical diagnosis, impeding successful therapy and intervention. Advanced materials are anticipated to revolutionize disease diagnostics. These materials include nanomaterials, biosensors, organic and inorganic compounds, polymers, and hybrid structures that can interact with biological molecules and signals in various ways.

We organize this special issue to give state-of-the-art findings in advanced materials for disease diagnostics. This special issue highlights the significance of materials in clinical diagnosis, including contributing subjects from inorganic, organic, and biological materials.

On the inorganic level, a research article from the Yun lab constructed smartphone-integrated colorimetric sensor array by using bluish-green fluorescent carbon quantum dots, for the fluorometric detection of dopamine in human plasma samples [1]. Noble metals always possess surface plasmon resonance under light irradiation, demonstrating potential in light-participated sensing and photo-thermal therapies. Janak L. Pathak et al. reviewed the significance of gold nanomaterials for oral cancer diagnosis and therapy in detail [2]. Further, Qiao et al. assembled gold nanoparticles to form water-in-oil colloidosomes and to encapsulate deproteinated serum for MALDI-TOF MS analysis. The colloidosomes-coupled matrix improved mass spectrometry performance, achieving accurate quantitation of free serum amino acids for the diagnosis of triple-negative breast cancer [3]. Particularly, the nanomaterial-based biosensor developing as a route toward in vitro diagnosis of early ovarian cancer was systematically reviewed by Kelong Ai lab [4].

Organic materials are desirable with tunable properties, in the optical sensing of disease-related biomarkers. Fengshou Wu et al. synthesized a new porphyrin polymer with donor-acceptorstructure, displaying intense absorbance in the near-infrared region with the maximum peak around at 850 nm [5]. Ding et al. outlined how organic persistent luminescence materials functions for in vivo bioimaging, focusing on their luminescence mechanisms and recent research progress, and the broad biological application prospects [6].

Advanced materials (particularly organic materials) usually serve as ideal supporting substrates, especially in electronic sensing. Liu et al. prepared hydrogel ionic diodes for label-free quantitative DNA detection and real-time monitoring of nucleic acid amplification [7]. Salzitsa Anastasova et al. fabricated a novel batteryless and wireless device suitable for the interrogation of pH and lactate in an embodiment model, with miniature biochemical sensing needles. Device readings were directly carried out by a smartphone, useful for assessing patient's health and potential port infection on demand [8]. Luo et al. designed a programmable molecular circuit called the Background-free isothermal circuital kit, to recognize multidrug-resistant bacteria. The kit included a molybdenum disulfide nanosheets-based fluorescence nanoswitch and non-specific suppression mediated by molecular inhibitors [9].

Other than single materials, various hybrid material complexes were also reported to enhance the diagnostic performance. Fang lab tailored the AgNWs-doped organic electrochemical transistors, integrating with multiplexed aptamer-antigen assays, to realize the signal amplification and simultaneous detection of biomarkers in raw sputum biospecimens from lung cancer patients [10]. Similarly, Gao et al. prepared hybrid materials by depositing Au on octahedral Pt-MOF for plasmonic effect and Schottky junction. The plasmonic hybrids enabled the enhanced hydrogenothermal therapy of rheumatoid arthritis [11]. Ye et al. reported human metabolite detection by surface-enhanced Raman spectroscopy, with focus on the enhancement induced by different plasmonic materials [12]. Liming Wu et al. employed tetrasulfide mesoporous silica-coated Nd-based rare-earth nanocrystals for tumor precise surgery navigation via NIR II fluorescence imaging [13].

Biological materials can interact with biological systems for diagnostic purposes. Biomaterials can be derived from natural sources, such as proteins, peptides, DNA, etc. Peptides are short chains of amino acids that can fold into specific conformations and interact with biological targets, such as receptors, enzymes, and antibodies. Wan et al. demonstrated the self-assembled peptide dendrimers with novel physicochemical properties, shedding light on theranostic applications [14]. Very recently, DNA has become a hot topic in material field, to create biomaterials with programmable structures and functions [7]. Two related articles presented by Ming Chen's lab [15] and Xianqiang Mi's lab [16] respectively, were included in this special issue to highlight their great promise in biomedical applications.

This special issue is also interested in the omic profiling and clinical discovery of novel biomarkers for disease diagnosis, *i.e.*, another kind of biomaterials. Biomarkers, including proteins [17], lipids [18], and RNA, were identified for the diagnosis of lung adenocarcinoma [19], prostate cancer [20], chronic HBV infection [21], *etc*. Exosomes are also clinically approved biomarkers for disease diagnosis, such as tumors. Baohong Liu et al. summarized recent advances in nanomaterials assisted exosomes isolation and analysis towards liquid biopsy [22], while Tony Y. Hu et al. focused on introducing new technologies for molecular diagnosis of cancer based on the tumor-derived exosome liquid biopsies [23].

Other functional materials, including hybrid nanodrugs for liver injury [24], cell membrane camouflaged biomimetic nanoparticles for tumor theranostics [25], and iron-oxide complexes for in vivo real-time red blood cell migration and microcirculation flow synergy imaging [26] were also included in this collection.

The special issue features invited and contributed reports within this broad interdisciplinary field. This collection of original reports and reviews in this special issue showcases the current state-of-the-art progress feeding this field, covering fundamental aspects and emerging applications of advanced materials to revolutionize disease diagnostics. We gratefully appreciate the support from the whole editorial team of Materials Today Bio and the reviewers. We would also like to take this opportunity to thank all authors who contributed to this special issue.

## References

[1] Chellasamy G., Ankireddy S.R., Lee K.N., Govindaraju S., Yun K. (2021). Smartphone-integrated colorimetric sensor array-based reader system and fluorometric detection of dopamine in male and female geriatric plasma by bluish-green fluorescent carbon quantum dots. Mater. Today Bio.

[2] Zhang Q., Hou D., Wen X., Xin M., Li Z., Wu L., Pathak J.L. (2022). Gold nanomaterials for oral cancer diagnosis and therapy: advances, challenges, and prospects. Mater. Today Bio.

[3] Han X., Li D., Wang S., Lin Y., Liu Y., Lin L., Qiao L. (2022). Serum amino acids quantification by plasmonic colloidosome-coupled MALDI-TOF MS for triple-negative breast cancer diagnosis. Mater. Today Bio.

[4] Yang Y., Huang Q., Xiao Z., Liu M., Zhu Y., Chen Q., Li Y., Ai K. (2022). Nanomaterial-based biosensor developing as a route toward in vitro diagnosis of early ovarian cancer. Mater. Today Bio.

[5] Li C., Luo Z., Yang L., Chen J., Cheng K., Xue Y., Liu G., Luo X., Wu F. (2022). Self-assembled porphyrin polymer nanoparticles with NIR-II emission and highly efficient photothermal performance in cancer therapy. Mater. Today Bio.

[6] Wu Z., Midgley A.C., Kong D., Ding D. (2022). Organic persistent luminescence imaging for biomedical applications. Mater. Today Bio.

[7] Xiong C., Li J., Li L., Chen L., Zhang R., Mi X., Liu Y. (2022). Label-free electrical monitoring of nucleic acid amplification with integrated hydrogel ionic diodes. Mater. Today Bio.

[8] Gil B., Lo B., Yang G.Z., Anastasova S. (2022). Smart implanted access port catheter for therapy intervention with pH and lactate biosensors. Mater. Today Bio.

[9] Hu X., Qin W., Yuan R., Zhang L., Wang L., Ding K., Liu R., Huang W., Zhang H., Luo Y. (2022). Programmable molecular circuit discriminates multidrug-resistant bacteria. Mater. Today Bio.

[10] Zhang R., Zhang J., Tan F., Yang D., Wang B., Dai J., Qi Y., Ran L., He W., Lv Y., Wang F., Fang Y. (2022). Multi-channel AgNWs-doped interdigitated organic electrochemical transistors enable sputum-based device towards noninvasive and portable diagnosis of lung cancer. Mater. Today Bio.

[11] Pan W., Li Z., Qiu S., Dai C., Wu S., Zheng X., Guan M., Gao F. (2022). Octahedral Pt-MOF with Au deposition for plasmonic effect and Schottky junction enhanced hydrogenothermal therapy of rheumatoid arthritis. Mater. Today Bio.

[12] Lu Y., Lin L., Ye J. (2022). Human metabolite detection by surface-enhanced Raman spectroscopy. Mater. Today Bio.

[13] Li J., Zhu F., Lou K., Tian H., Luo Q., Dang Y., Liu X., Wang P., Wu L. (2022). Tumor microenvironment enhanced NIR II fluorescence imaging for tumor precise surgery navigation via tetrasulfide mesoporous silica-coated Nd-based rare-earth nanocrystals. Mater. Today Bio.

[14] Xie F., Li R., Shu W., Zhao L., Wan J. (2022). Self-assembly of Peptide dendrimers and their bio-applications in theranostics. Mater. Today Bio.

[15] Yu L., Yang S., Liu Z., Qiu X., Tang X., Zhao S., Xu H., Gao M., Bao J., Zhang L., Luo D., Chang K., Chen M. (2022). Programming a DNA tetrahedral nanomachine as an integrative tool for intracellular microRNA biosensing and stimulus-unlocked target regulation. Mater. Today Bio.

[16] Wang C., Xu Y., Li S., Zhou Y., Qian Q., Liu Y., Mi X. (2022). Designer tetrahedral DNA framework-based microfluidic technology for multivalent capture and release of circulating tumor cells. Mater. Today Bio.

[17] Yi W., Qiao T., Yang Z., Hu L., Sun M., Fan H., Xu Y., Lv Z. (2022). The regulation role and diagnostic value of fibrinogen-like protein 1 revealed by pan-cancer analysis. Mater. Today Bio.

[18] Jiao B., Zhou W., Liu Y., Zhang W., Ouyang Z. (2022). In-situ sampling of lipids in tissues using a porous membrane microprobe for direct mass spectrometry analysis. Mater. Today Bio.

[19] Xu X., Cui J., Wang H., Ma L., Zhang X., Guo W., Xue X., Wang Y., Qiu S., Tian X., Miao Y., Wu M., Yu Y., Xu Y., Wang J., Qiao Y. (2022). IGF2BP3 is an essential N(6)-methyladenosine biotarget for suppressing ferroptosis in lung adenocarcinoma cells. Mater. Today Bio.

[20] Dong L., Du X., Lu C., Zhang Z., Huang C.Y., Yang L., Warren S., Kuczler M.D., Reyes D.K., Luo J., Amend S.R., Xue W., Pienta K.J. (2022). RNA profiling of circulating tumor cells systemically captured from diagnostic leukapheresis products in prostate cancer patients. Mater. Today Bio.

[21] Xun Z., Yao X., Zhu C., Ye Y., Wu S., Chen T., Zeng Y., Lin C., Yang B., Ou Q., Liu C. (2022). Proteomic characterization of the natural history of chronic HBV infection revealed by tandem mass tag-based quantitative proteomics approach. Mater. Today Bio.

[22] Fang X., Wang Y., Wang S., Liu B. (2022). Nanomaterials assisted exosomes isolation and analysis towards liquid biopsy. Mater. Today Bio.

[23] Li L., Zhang L., Montgomery K.C., Jiang L., Lyon C.J., Hu T.Y. (2023). Advanced technologies for molecular diagnosis of cancer: state of pre-clinical tumor-derived exosome liquid biopsies. Mater. Today Bio.

[24] Liu M., Huang Q., Zhu Y., Chen L., Li Y., Gong Z., Ai K. (2022). Harnessing reactive oxygen/nitrogen species and inflammation: nanodrugs for liver injury. Mater. Today Bio.

[25] Zhu L., Zhong Y., Wu S., Yan M., Cao Y., Mou N., Wang G., Sun D., Wu W. (2022). Cell membrane camouflaged biomimetic nanoparticles: focusing on tumor theranostics. Mater. Today Bio.

[26] Ye F., Zhang B., Qiu L., Zhang Y., Zhang Y., Zhang J., Zhao Q., Lu L., Zhang Z. (2022). In vivo real-time red blood cell migration and microcirculation flow synergy imaging-surveyed thrombolytic therapy with iron-oxide complexes. Mater. Today Bio.

